# Mini PCNL Over Standard PCNL: What Makes it Better?

**DOI:** 10.1055/s-0040-1701225

**Published:** 2020-02-12

**Authors:** Bikash Bikram Thapa, Vikram Niranjan

**Affiliations:** 1 Department of Surgery, Nepal Army Institute of Health Sciences, Kathmandu, Nepal; 2 Health Research Institute/Graduate Entry Medical School, University of Limerick, Limerick, Ireland

**Keywords:** nephrolithiasis, percutaneous nephrolithotomy, mini-PCNL

## Abstract

The incidence of small- and medium-size renal stones is rising. Stone clearance, bleeding, urine leak, and infectious complications are major concerns for urologists. They can choose the best technique from a list of armamentarium available. Minimally invasive approach like percutaneous nephrolithotomy (PCNL) has significantly influenced renal stone management since 1976. Miniaturization of the instruments innovate more effective and safer alternatives for urolithasis management. The outcome of mini-PCNL is explored and compared with standard PCNL in this review. Original research articles were reviewed using a systematic approach (keyword electronic database search). Duplicates were excluded in each step and 19 original articles out of 156 hits were analyzed. Mini-PCNL has significantly less bleeding complications and hospital stay. There were no significant difference in stone free rate between mini-PCNL and standard PCNL. The stone-free rate and complications rates were less dependent on the technique of puncture, tract dilatation, and energy used to fragment stones. The total operative time became slightly longer in mini-PCNL attributed to the sheath size and stone fragments retrieval. We found that mini-PCNL is as effective as standard PCNL with fewer complications. Stone burden is the key factor responsible for overall stone-free rate. However, the recommendation is limited by quality of study and the sample sizes.

Urolithiasis means a calculus anywhere in the urinary tract, whereas nephrolithiasis refers to a calculus in kidney. There has been increase in incidence and prevalence of nephrolithiasis globally and is unique to climate and the socioeconomic status. 1 2 There is paradigm shift in the management of the nephrolithiasis with the invention of the minimally invasive endourological procedure. The international guidelines recommend percutaneous nephrolithotomy (PCNL) as the first line of treatment for renal stones more than 20 mm in size. Whereas for stones of size 10 to 20 mm the treatment options can be shock wave lithotripsy (SWL), PCNL, or retrograde intrarenal surgery (RIRS). 3 4 The procedure PCNL has evolved since 1976 and has undergone many modifications and refinements in the techniques and the instruments to achieve maximum stone clearance with minimal complications. One of them is miniaturizing the access sheath. Standard PCNL is done with sheath size of 24 to 30 F, whereas the mini-PCNL/miniperc is done with sheath size 14 to 20 F. 5 A meta-analysis 6 published in 2015 mentioned that the size of PCNL access sheath matters. Mini-PCNL is safer and had equal efficacy rate for management of renal stones. We are revisiting the mini-PCNL, reviewing and comparing its success in management of renal stones.

**Methodology**

PubMed, Google Scholar, Cochrane, and Embase were searched for “Mini PCNL” and/or “Miniperc” and paired with “Outcome” and “Complication.” The search resulted in 156 related articles. The first 67 articles that did not match with the key word and had other description like ultra-mini and super-mini were excluded. Research titles that evaluate or compare mini-PCNL over standard PCNL for nephrolithiasis only were selected for review.

## Inclusion Criteria

The inclusion criteria are as follows:

▪ Original research article
▪ Randomized or nonrandomized comparison between mini-PCNL and standard-PCNL
▪ Comparing different techniques of mini-PCNL
▪ Outcomes measured in operative time, morbidities, length of hospital stay, and stone-free rate (SFR)
▪ Studies managing renal calyceal and pelvic stones

**Fig. 1:**
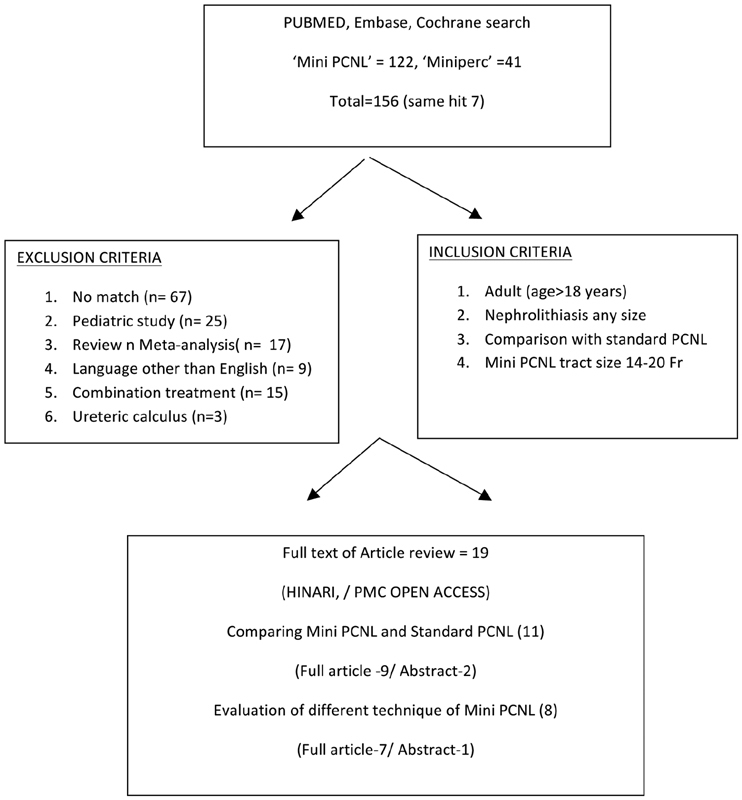
Flowchart for study design-—duplicates in each step were excluded and final original articles were included for review.

After excluding the original articles that do not meet the inclusion criteria, 19 articles were selected for review. Ten original articles comparing mini-PCNL with standard PCN and seven original articles evaluating different techniques of mini-PCNL were included. The safety of the procedure (mini-PCNL) was compared over standard PCNL in terms of operative time, drop in hemoglobin, blood transfusion rate, infectious complications, and length of hospital stay. The efficacy was explored in terms of the SFR ( Fig. 1 ).

## Results

### Mini-PCNL Is Safer with Equal Efficacy with Standard PCNL

Jackman performed the first mini-PCNL in an adult patient, using a small access sheath (13 F) with a miniature instrument (6.9 F/7.2 F ureteroscope or 7.7 F pediatric cystoscope) in 1997. The result of the very first mini-PCNL in seven adult patients had encouraging result with SFR of 89%. Miniperc quickens the recovery after PCNL with lesser operative time (60 ± 19 min), hemoglobin drop (1 ± 0.6 g %), morbidities (4.7%), and lesser hospital stay (2.8 ± 1 day). 7

Out of 11 comparative studies done for min-PCNL and standard PCNL, 7 were prospective nonrandomized, 2 were prospective randomized, and 2 were retrospective studies. A study published as early as 2006 by Giusti et al 8 publishes retrospective data with a self-explanatory title “Mini-perc? No thank you!” Since then many studies have compared these two modalities and it is still a subject of interest till date. The studies were heterogeneous in size of stone, access sheath, type of endoscopes, type of lithotripsy, and use of PCN tube drain.

One of the largest series of prospective studies by Li et al 9 published comparable SFR mini- and standard PCNL with significantly lesser rate of blood transfusion in mini-PCNL group (1.1% vs. 6.9%). He failed to demonstrate reduced invasiveness of the smaller tract size of mini-PCNL in molecular level and the complication rates were comparable. A higher rate of tubeless procedure ( *p* < 0.001), lesser hospital stay (3.2 ± 0.8 vs. 4.8 ± 0.6 days, *p* ≤ 0.001) was added to the advantage of reduced drop in hemoglobin (0.8 ± 0.9 vs. 1.3 ± 0.4 g, *p* = 0.01) by Mishra et al. 10 Similar result was obtained by other prospective randomized studies with a twice larger sample size. 11 12 The tubeless PCNL was done ranging from 50 to 95% of the sample size in mini-PCNL. There was no statistically significant difference in total operative time between mini-PCNL and standard PCNL (mini-PCNL 24–155 minutes and standard PCNL 25–103 minutes). The reported SFR for stone burden 10 to 30 mm was as high as 96% in mini-PCNL and 100% in standard PCNL ( Table 1 ).

**Table 1:** Comparison of mini-PCNL versus standard PCNL

Studies, Year	Number N = mPCNL vs. sPCNL	Stone size (mm)	Sheath size (F)	Operative time (minute)	Drop in Hb (g/dL)	Hospital stay (days)	Tubeless procedure	Intraoperative complications (%)	SFR (%)	Analgesic use	Level of evidence	Quality of study *
Giusti et al 8 2006 **Prospective**	40 vs. 67	28 vs. 31	13 vs. 30	155.5 ± 32.9 106.6 ± 24.4	4.49 ± 3.10 6.31 ± 4.29	3.05 ± 1.69 vs. 5.07 ± 2.15	**14 vs. 13**	NA	77.4 vs. 94	5.53 ± 1.14 vs. 6.36 ± 1.67	3b	6
**P value- NA**												
Li et al 9 2010 **Prospective**	93 vs. 72	28.6 vs. 30.2	14–18 vs. 26	87.6 vs. 64.5	8.8 vs. 16.3	6.3 vs. 6.3	**NA**	NS	83.9 vs. 87.5	NA	3b	6
**P value**		0.223		0.006	0.002	0.94	**NA**	NS	0.25	NA		
Knoll et al 15 2010 **Prospective**	25 vs. 25	18 ± 8 vs. 23 ± 9	18 vs. 26	48 ± 17 vs. 57 ± 22	NA	3.8 ± 28 *s* 6.9 ± 3.5	**All tubeless**	12 vs. 20	96% vs. 92%	3 ± 3 vs. 4 ± 3	3b	6
**P value**		0.042		NS	NA	0.021		NS	NS	0.04		
Cheng et al, 18 2010 **RCT**	72 vs. 115	All types	16 vs. 24	90–135 vs. 77–119	0.53 ± 0.79 vs. 0.97 ± 1.42	7.3 vs. 7.5	**NA**	No significant difference	70–90	88.7 ± 40.6 Vs. 94.3 ± 37.2	3b	7
**P value**		>0.05		<0.05	<0.05	>0.05	NA	>0.05	NS	>0.05		
Zhong et al 24 2011 **RCT**	29 second 25	117 vs. 108 mm ^3^	16 vs. 25	116 vs. 103	3.2 vs. 3.5	9.8 vs. 7.1	NA	37.9 vs. 52	89 vs. 68	NA	2b	7
**P value**		NS		0.052	0.09	0,07	NA	0.3	0.03			
Mishra et al, 10 2011 **Prospective study**	27 vs. 28	14.7 ± 0.3 vs. 14.9 ± 0.6	18.2 ± 2 vs. 26.8 ± 2	45.2 ± 12.6 vs. 31 ± 16.6	0.8 ± 0.9 vs. 1.3 ± 0.4	3.2 ± 0.8 vs. 4.8 ± 0.6	21 vs. 4	3 vs. 10	96 vs. 100	55.4 ± 50 vs. 70.2 ± 52	3b	7
**P value**		0.8		0.0008	0.01	0.001	0.001	NS	NS	NS		
**Lange** 13 **, 2017**	29 vs. 27	10–35	16.5 vs. 30	NA	0.02	NA	NA	NA	NA	NA	3b	6
**P value**				NS	0.02	NS	NA	NS	NS	NA		
Elsheemy et al. 14 **2018 Retrospective**	378 vs. 151	37.7 ± 2.21 vs. 37.7 ± 2.43	18 vs. 30	68.6 ± 29.09 vs. 60.49 ± 11.38	Blood transfusion 3.7% vs. 7.9%	2.43 ± 1.46 vs. 4.29 ± 1.28	248 vs. 7	30 vs. 31 (7.9% vs. 20.5%)	First session 86.8% vs. 90.7%	NA	3b	6
**P value**		0.3		0.4	0.04	<0.001	<0.0001	<0.001	0.2			
Kukreja et al, 12 2018 **Prospective**	61 vs. 62	20.6 ± 3.47 vs. 21.5 ± 3.53	16.5/17.5 vs. 22/24	25.46 ± 11.9 vs. 24.68 ± 12.45	0.87 ± 0.72 vs. 1.48 ± 0.83	**NA**	58 vs. 48	NA	93 vs. 91.9	0.3 ± 0.54 vs. 0.43 ± 0.65	3b	7
**P value**		0.1		0.3	<0.001	NA	0.01		NS	NS		
Güler A et al 25 2019 **RCT**	46 vs. 61	>2 0		∓	∓	∓	∓	∓	76.5% vs. 71.7%,	NA	2b	7
**P value**				0.012	NA	0.01	NA	0.31	NS	NA		

Abbreviations: SFR, stone-free rate; NS, not significant; NA, not available.

* Newcastle–Ottawa Scale (score 0– 9).

Complex stone burden with stone size of 10 to 35 mm can be effectively managed with less blood loss in mini-PCNL technique. 7 13 Elsheemy et al 14 managed all type of stones (staghorn, multiple calyceal, simple) using either mini-PCNL (378) or standard-PCNL (151). Mini-PCNL had longer operative time (68.6 ± 29.09 vs. 60.49 ± 11.38 min; *p* = 0.434); shorter hospital stay (2.43 ± 1.46 vs. 4.29 ± 1.28 days), and higher rate of tubeless PCNL (75.1% vs. 4.6%). Complications were significantly higher in standard PCNL (7.9% vs. 20.5%; p < 0.001) with higher rate of blood transfusion (7.9% vs. 3.7% with *p* = 0.041). Complex stone burden required multiple tracts or multiple session of PCNL. 14 15 Mini-PCNL in complex stone burden had lesser overall SFR in mini-PCNL (86.8% in the first session and 89.9% after the second session) than the standard PCNL (90.7% in the first and 96% after the second session). 14 Most of the studies found no significant difference in postoperative complication rate and analgesic use between two procedures ( Table 1 ). Postoperative pain and fever, bleeding, and urine leak were common complications in both group. Tubeless mini-PCNL causes significantly less postoperative pain and less pain medication use. 8 12 15 The overall complication rate after mini-PCNL ( *n* = 1,000) was reported to be 20.1%, out of which 7.4% were Clavien grade I, 8.8% were grade II, and 3.5% were grade III complications, but no grade IV or V complications were found. 16

### Stone Burden as Key Factor of Safety and Efficacy

A total of 10,000 mini-PCNL was performed between 1992 and 2011 by Zeng et al, 17 where 5,761 (41.2%) were simple calyceal stones and 8,223 (58.8%) were complex calyceal stones. The stone burden was lower in simple calyceal stones, 1018.6 mm vs. 1763.0 mm ( *p* < 0.05). Patients with simple stones had significantly shorter operative time, less hemoglobin drop, and higher SFR (77.6% vs. 66.4%) after a single session of mini-PCNL ( *p* < 0.05). The differences diminished after relook and/or auxiliary procedures (86.7% vs. 86.1%, P > 0.05). The complication rate (17.9% vs. 19.0%) and blood transfusion rate (grade II) (2.2% vs. 3.2%) were similar in both groups (P > 0.05). Renal vascular embolizations (grade III) were significantly higher with complex stone burden ( *p* < 0.05). SFR was less with multiple stones ( *p* = 0.018) and large stone burden > 2 cm ^2^ ( *p* = 0.026). 14 18 In comparison to standard PNCL, mini-PCNL was more efficient to manage multiple caliceal stones (SFR 85.2% vs. 70%, *p* < 0.05) and equally efficient for simple renal pelvis stone and staghorn stones adjusted for number of tract and PCNL session. 18

### Role of Instrument and Equipment

Comparison of mini-PCNL and standard PCNL were not adjusted for technical aspect of procedure (image guidance for puncture, type of dilator, size of sheath, type of lithotripter, etc.). Few studies had compared role of technical factors in outcome of mini-PCNL only. Arslan 1 found no significant difference in SFR (82.1% vs. 79.5%, *p* = 0.285) in between single step metal coaxial dilator and serial Amplatz dilator during mini-PCNL. The rate of perioperative complication was similar. Though the fluoroscopy time and the total hospital stay were longer ( *p* < 0.001) in metal sheath group it was more cost effective than Amplatz sheath group. Mini-PCNL had the same safety and efficacy in management of low stone burden (STONE scores 5–6) among all three approaches of calyceal puncture (viz. fluoroscopy vs. ultrasound vs. combined fluoroscopy and ultrasound). Fluoroscopic guidance and combined (fluoroscopic and ultrasound) guidance resulted in higher SFR (89.4% vs. 90.2% vs. 69.8%, *p* = 0.002) than ultrasound guidance only in renal stone with complex burden (stone scores 7–8). Combined guidance had significantly longer access time ( *p* = 0.003) and no difference in complication rate and hospital stay. 19

The operative time, drop in mean hemoglobin, complication rates, postoperative pain, and SFR were similar for type of energy used for lithotripsy (p > 0.2). Similar safety and efficacy were noted in management of comparable two groups of renal stone using laser and ultrasound by mini-PCNL procedure. The SFR was higher in laser lithotripsy group than ultrasound group, though it was not statistically significant (81.8% vs. 68.2% and *p* = 0.296). 20 Stone migration was lower and fragment removal was effective with laser lithotripsy. Action required for stone fragments retrieval was less in laser lithotripsy group than pneumatic lithotripsy (10% vs. 37%, *p* = 0.002) 21

## Discussion

Since its first use in 1997 mini-PCNL has been an increasingly popular alternative for the management of the renal stones due to its higher safety profiles. Lahme recommended mini-PCNL to treat all kinds of upper urinary tract calculi greater than 10 mm in diameter and it is regarded as a treatment alternative to flexible ureterorenoscopic lithotripsy (URSL), shock wave lithotripsy (SWL), and conventional PCNL. 22 For complete stone clearance the use of auxiliary procedure like second PCNL, SWL, and URSL are often necessary.

Mini-PCNL had significant advantage over standard PCNL in terms of reduced bleeding, leading to a higher chance of tubeless procedure (75–80%) and reduced hospital stay (2.43–4.5 days) ( Table 1 ). The longer operative time was attributed to stone burden and type of energy used for lithotripsy. Laser lithotripsy is efficient but takes longer time than pneumatic lithotripsy ( *p* < 0.001). 20 21

The overall complication doesn't differ between mini-PCNL and standard PCNL. Untreated preoperative urinary tract infection, high perfusion pressure, longer operative time, toxin absorption and pelvicalyceal system perforation, and poor drainage of the pelvicalyceal system after surgery are responsible for increase in complications. 22 The nephrostomy tube placed at the end of the procedure has several advantages. It allows uninterrupted drainage of urine from kidney, tamponade effect on the renal access tract, and allows for a “second look” surgery if needed. Tubeless PCNL had advantage for less postoperative pain and early discharge. 23

## Conclusion

Mini-PCNL is as effective as standard PCNL with less blood loss in small and medium size stone (10–30 mm). Stone burden is the key denominators for optimal stone free rate. Even a complex stone burden is amenable to mini-PCNL. Most of the comparative studies have small sample size and are nonrandomized. The effect of patient position in outcome is inconspicuous. The comparisons were not adjusted for different technical details like puncture guidance, type of dilators, tract size, and lithotripsy. Multicenter randomized studies with subgroup analysis can draw more robust evidence in the field.
